# Strychnine Poisoning

**Published:** 1891-01-24

**Authors:** 


### STRYCHNINE POISONING.

A young lady at Riga, took soon after a meal a dose of five grains of strychnine with suicidal intention. Dr. Blumenbach saw her about half an hour later, when he found her in a state of complete rigidity, while the lightest touch sufficed to excite convulsions. Breaking a tooth he succeeded in introducing the tube of a stomach pump, and washed out the stomach until fifteen litres of water had been used without producing any effect on the spasms. A subcutaneous injection of 0 03 grm. (⅕ gr.) of morphia with the inhalation of chloroform to narcosis was followed by great and rapid improvement, and ten or twelve days afterwards she had completely regained her health. The failure of the stomach pump to afford any relief is in accordance with the statement of Binz, that strychnine performs the entire circle of the vascular system within five minutes, and that attempts at evacuation of the stomach by mechanical means, only increase the muscular spasms.

